# Is it worth investing in mental health promotion and prevention of mental illness? A systematic review of the evidence from economic evaluations

**DOI:** 10.1186/1471-2458-8-20

**Published:** 2008-01-22

**Authors:** Ingrid Zechmeister, Reinhold Kilian, David McDaid, the MHEEN group

**Affiliations:** 1 Post-doc researcher at the Ludwig Boltzmann Institute for Health Technology Assessment, Garnisongasse 7/20, 1090 Vienna, Austria; 2 Senior lecturer at the University of Ulm, Department of Psychiatry II, BKH Guenzburg, Ludwig-Heilmeyer-Str. 2, D-89312 Guenzburg, Germany; 3 Research fellow at the LSE Health and Social Care and European Observatory on Health Systems and Policies, London School of Economics and Political Science, Houghton Street, London, WC2A 2AE, UK

## Abstract

**Background:**

While evidence on the cost of mental illness is growing, little is known about the cost-effectiveness of programmes in the areas of mental health promotion (MHP) and mental disorder prevention (MDP). The paper aims at identifying and assessing economic evaluations in both these areas to support evidence based prioritisation of resource allocation.

**Methods:**

A systematic review of health and non health related bibliographic databases, complemented by a hand search of key journals and analysis of grey literature has been carried out. Study characteristics and results were qualitatively summarised. Economic evaluations of programmes that address mental health outcome parameters directly, those that address relevant risk factors of mental illness, as well as suicide prevention interventions were included, while evaluations of drug therapies were excluded.

**Results:**

14 studies fulfilled the inclusion criteria. They varied in terms of topic addressed, intervention used and study quality. Robust evidence on cost-effectiveness is still limited to a very small number of interventions with restricted scope for generalisability and transferability. The most favourable results are related to early childhood development programmes.

**Conclusion:**

Prioritisation between MHP and MDP interventions requires more country and population-specific economic evaluations. There is also scope to retrospectively add economic analyses to existing effectiveness studies. The nature of promotion and prevention suggests that innovative approaches to economic evaluation that augment this with information on the challenges of implementation and uptake of interventions need further development.

## Background

Increasing evidence about the high social and economic costs of poor mental health has contributed to a growing recognition of the need to promote positive mental health and wellbeing, as well as to prevent the onset of mental illness [1-3]. This is supported by data showing that promotion and prevention strategies reduce the individual and social impacts of poor mental health [4]. Thus, these interventions may not only be effective but potentially cost-effective in an economic sense.

One in four individuals can expect to experience mental health problems during their lifetimes [5]. Conservatively the costs of poor mental health have been estimated to account for between 3% and 4% of GDP in developed countries [6]. A number of long term longitudinal studies indicate that untreated mental health and behavioural problems in childhood, in particular, can have profound longstanding social and economic consequences in adulthood, including increased contact with the criminal justice system, reduced levels of employment and often lower salaries when employed, and personal relationship difficulties [7-10]. Yet there is a growing body of literature that provides some (typically short term) evidence on the effectiveness of a range of prevention programmes which address known or at least assumed risk factors for mental disorders [11-20]. There is also some evidence on the effectiveness of programmes focussing on the early detection and early intervention for severe mental disorders, particularly depression and schizophrenia [21-24].

In the context of increasing budget constraints and rising demands for evidence-based health spending, evidence on effectiveness alone is insufficient for policy making; in addition to knowing what works and in what context, information on the economic costs and consequences of any intervention is required. Given the inevitable constraints on funds available for all public sector interventions, economic evaluations are increasingly used as one key input to decisions on how to allocate resources to various health system actions, most notably for assessing pharmaceuticals, medical devices and procedures [25]. More recently, arguments have also been put forward to examine the economic case of all areas of public health and health promotion [26] and while some studies have been conducted in these areas, such evaluations remain few compared with health care interventions [27].

It is therefore no surprise that the use of economic evaluations in the field of mental health care generally continues to grow [28], but little has previously been reported on the extent to which the cost-effectiveness of mental health promotion (MHP) and mental disorder prevention (MDP) programmes has been considered and, if so, what form these evaluations take. This paper aims to systematically collate relevant evidence to help address the question as to whether it is worth investing in MHP and MDP interventions from an economic perspective. It seeks to identify those areas in which economic evaluations have most commonly been used, and reports on the economic techniques used. It also looks at the extent to which available economic studies focus solely on the prevention of mental health disorders or also have sought to assess the cost effectiveness of the promotion of improved mental well-being.

## Methods

A systematic review protocol was developed in line with recommended guidelines [29-31] in order to identify economic evaluations in the areas of MDP and MHP. A range of bibliographic databases, MEDLINE/PUBMED, Embase, Cinahl, Psychinfo, Psyndex, Econlit, ERIC and NHS EED were included in the protocol. This was complemented by a hand search of key journals, as well as an internet search for grey literature, including governmental reports and academic working papers. Precise search terms were dependent on the database used, but all included core medical subject headings and title/abstract phrases including 'mental disorder', 'mental illness', '(primary) prevention', 'health promotion', 'preventive measures', 'occupational health', 'workplace health promotion' and 'suicide prevention'. In addition to the use of the Medical Subject Heading 'costs and cost analysis', other economic terms used were 'economics', 'cost effectiveness', 'cost utility', 'cost benefit', 'cost consequences' and 'economic evaluation'.

Considering the fragmented pattern of scientific evidence in the MHP and MDP fields a pragmatic approach to defining inclusion and exclusion criteria was required. Firstly, those economic evaluations of mental health promotion or prevention interventions were included that addressed either mental well-being or mental disorder as an outcome parameter directly (e.g. changes in a measure of happiness or a depression scale). Secondly, studies which focused on preventing outcomes specifically recognised as well-known risk factors for future mental disorder were included (e.g. behavioural problems in children). Additionally, we included papers on suicide prevention, although suicide is not always related to mental health problems.

While the definition of mental disorders was based on the ICD classification, studies which evaluated programmes on alcohol and drug dependency were excluded. In respect of prevention we restricted the review to studies on primary and secondary prevention alone as defined by the WHO [32]. Thus, we included evaluations of early detection to lower the rate of established cases of mental disorders, while tertiary prevention which would include much use of drug therapy was excluded. Furthermore, with respect to economic evaluation, we took into account methodological discussions which suggest that for public health interventions the definition of economic evaluation must be broadened so as to include methods such as econometric studies of regulatory, fiscal or legislative change. In addition, we wanted to ensure that studies which combined qualitative evidence on impact, such as individual satisfaction rates, alongside economic data would be included in our review [33]. Thus, the review protocol design went beyond conventional economic evaluations to identify other relevant economic analyses, including cost consequence analysis where costs and effectiveness measures are not synthesised into a single ratio. However, studies that only addressed costs such as cost-of-illness studies were excluded.

There were no date restrictions imposed on the bibliographic database search, but our analysis was limited to studies in English and German. Study selection was initially on the basis of study abstract, with data extraction and assessment conducted independently by two reviewers based on available checklists [25,34]. According to suggested methodological standards [35] quantitative results in monetary terms were converted into the single currencies of US$ and Euros by using GDP Purchasing-Power-Parities. Additionally, they were adjusted for price levels referring to the year 2006.

## Results

**Figure 1:**
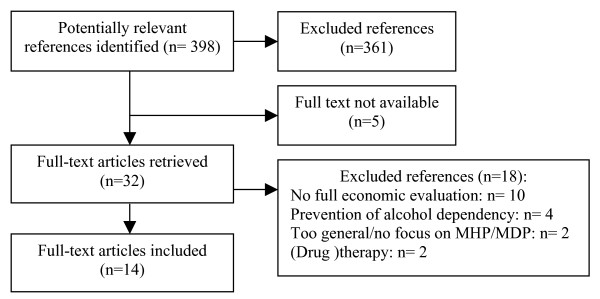
Flow diagram of study selection process.

This search process resulted in 398 hits. 361 papers were excluded on the basis of abstract alone. From the 37 remaining studies, 5 could not be obtained, leaving 32 studies, 18 of which did not meet all inclusion criteria, leaving 14 studies in our analysis (see Figure 1; for a full reference list of excluded studies see appendix).

Compared to the economic evaluation of medical interventions for mental illness (e.g. pharmaceuticals), the number of available studies that evaluate MHP or MDP interventions from an economic perspective is low. The broad and fragmented nature of promotion and prevention interventions meant that our hand and internet search were however able to identify 8 of the 14 studies, as they did not appear in the largely medical databases. Table 1 provides a summary of the study characteristics and main results.

**Table 1:** Study characteristics and results

**Source**	**Evaluation type**	**Country**	**Intervention**	**Source effectiveness data**	**Type of data**	**Time horizon**	Results*
*Programmes to prevent depression/sucide*							

Smit et al. (2006) [38]	CEA	Nether-lands	Minimal contact-therapy for primary prevention of depression	Pragmatic RCT	Primary data	1 yr	80% probability of cost-effectiveness if WTP per case avoided is below US$ 23,000
Petrou et al. (2006) [39]	CEA	UK	Home visiting therapist f. primary prevention of post-natal depression	Pragmatic RCT	Primary data	18 months	70% probability of cost-effectiveness if WTP per depressive month avoided is below US$ 1,800
Lynch et al. (2006) [46]	CEA	USA	CBT for high at risk teens for depression	RCT	Secondary data	1 yr	US$ -14 to US$ 24 per DFD; US$ -12,200 to US$ 3,400 per QALY
Valenstein et al. (2001) [47]	CUA	USA	Various types of Screening for depression	Secondary literat./not specified; Meta-analysis of RCTs	Simulation/modelling	Life-time	Cost utility ratios unfavourable; 1-time screening compared to no screening lowest ICUR(on average US$ 47,000 per QALY)

Sari et al. (2007) [49]	CBA	USA	General education and peer support to prevent suicide in high-school	Secondary literature/not specified	Simulation/modelling	1 yr	Net benefit: US$ 21 million and US$ 32 million respectively; ratio: US$ 2.36:1 and US$ 4.3:1
Zaloshnja et al. (2003) [44]	CUA/CBA	USA	Lay people training for crisis-support	Prospective observational trial	Secondary data	10 yrs	Benefit-cost ratio: 47:1; ICUR: US$ 460 per QALY
Appleby et al. (2000) [42]	CEA	UK	Education for health professionals to assess and manage suicidal patients	Prospective observational trial	Primary data	1 yr	US$ 6,200 per LYG; US$ 183,000 per suicide prevented
Byford et al. (1999) [41]	CCA	UK	Social work intervention for adolescents with risk for self-harm	Pragmatic RCT	Primary data	5 months	Intervention not more effective and not more costly
Rutz et al. (1992) [45]	CBA	Sweden	Educational programme for GPs to detect depression	Prospective observational trial	Secondary data	Life-time	Net benefit: US$ 37 million

*Programmes that address overall risk factors for mental disorders*							

Wiggins et al. (2004) [40]	CCA	UK	Post-natal support for young mothers in deprived city areas	Pragmatic RCT	Primary data	18 months	Interventions not more effective and not cost saving
McAuley et al. (2004) [43]	CEA, CCA	UK	Home start support for young families	Prospective observational trial	Primary data	11 months	Intervention not more effective and more costly

Schweinhart (2005) [48]	CBA	USA	Early child development programme (ECD)	Pragmatic RCT	Simulation/modelling	40 yrs	US $ 19.81 return per invested US$
Lynch (2004) [36]	CBA	USA	ECD	Several pragmatic RCTs	Simulation/modelling	45 yrs	After 17 yrs: budget benefits outweigh costs; benefit-cost ratios: US$ 4.01 to 9.27 per $ invested
Aos et al. (2004) [37]	CBA	USA	Several type of childhood and adolescent support programmes	Systematic review of RCTs	Simulation/modelling	Life-time	From net-benefit of US$ 33,100 to net costs of US$ 52,000 depending on programme

CBA: Cost benefit analysis; CCA: cost-consequence analysis; CEA: Cost effectiveness analysis; CUA: Cost utility analysis; DFD: depression free day; ICUR: Incremental cost utility ratio; LYG: Life year gained; QALY: quality adjusted life years; RCT: Randomised controlled trial; WTP: willingness to pay; * original results have been converted into US$ according to GDP-PPP where required and inflated to 2006 price levels

Half of these studies evaluated programmes in three European countries (the UK, Netherlands and Sweden) with the remainder being in the US. Some of these US based evaluations, in fact covered a number of programmes [36,37]. One made use of effectiveness results from a vast number of different US early childhood development (ECD) interventions and adolescent support programmes [37]. In terms of (effectiveness) evaluation activity, common with many health promoting or public health interventions, this seemed to be higher in the USA than in Europe (at least in the field of ECD). The majority of economic evaluations applied conventional economic techniques such as cost benefit, cost effectiveness, cost utility or cost consequence analysis. Some studies included simulation models in order to look at projected long term costs and benefits.

While six studies were conducted prospectively alongside the collection of primary data [38-43], eight economic evaluations were either based on the retrospective secondary analysis of data resulting from experimental or quasi-experimental studies [44-46], or developed simulation models using effectiveness data obtained through systematic and non-systematic reviews [36,37,47-49]. Although in the majority of studies the study design for measuring effectiveness was based on randomly selected intervention and control groups, due to the nature of these interventions double-blinding was not possible. As in any other case of preventive intervention evaluations, this raises the potential of bias in outcome measurement. Moreover, in some cases the study design was a controlled observational trial where the intervention group was compared to a non-randomised control group. This again is sub-optimal with respect to evaluating the causal relationships between an intervention and outcomes (see table 1).

While the evaluations identified were diverse in terms of type of intervention, a high proportion of the studies dealt with measures to prevent depression, sometimes with a secondary objective of preventing suicide. The remainder focused on addressing some of the overall risk factors for mental disorders. No economic evaluations were found of interventions that address mental well-being rather than mental illness as an outcome parameter. In the following section, the main characteristics of studies are briefly presented.

### Programmes to prevent depression or suicide

Smit et al. (2006) [38] in the Netherlands evaluated the cost-effectiveness of minimal-contact psychotherapy as a preventive measure for individuals at high risk of depression. At 12 months the incidence rate ratio of depression was 0.65 when comparing the intervention (11.9) and usual care groups (18.3). At a cost-effectiveness threshold of US$ 23,000 (€ 20,000) per case avoided, a 70% to 80 % probability (depending on the costs included) that the intervention would be cost-effective compared to standard actions was shown. Additionally, there was a 40% to 60% probability that the intervention would be dominant compared to standard care (i.e. better outcome at a lower cost). However, these calculations are based on a rather short time-horizon of one year and the trial design does not guarantee an unbiased evaluation. As the authors remark, the results cannot be reliably transferred to other countries because of the differing health care system contexts.

In England, Petrou et al. (2006) [39] evaluated a preventive intervention targeted at women who were at high risk of developing postnatal depression. Based on a pragmatic randomised controlled trial they found a non-significant increase in depression-free-months, as well as a non-significant increase in health and social care costs in the intervention group. At a willingness to pay threshold of US$ 1,800 (€ 1,500) per prevented month of depression, the probability that the intervention is cost-effective was reported to be 70%. The non-significant nature of effectiveness data meant that this probability did not exceed 80% even at substantially higher willingness to pay thresholds.

Lynch et al. (2005) [46] evaluated the cost effectiveness of cognitive-behaviour therapy for high-risk teenagers with depressive parents over a one year period. In the year after the intervention, intervention participants reported significantly more depression free days than the control group (301 versus 248). The authors argued in favour of the intervention based on the fact that cost-effectiveness ratios are within the same range as those for accepted depression treatments in the US, with the reported cost per quality adjusted life year (QALY) remaining below the threshold of US $ 50,000 (€ 45,000) per QALY gained. However, the long-term effects of the intervention were not analysed and the intervention group was rather small which does not allow the results to be generalised.

Valenstein et al. (2001) [47] evaluated alternative strategies of screening for depression among adults in the US. They assumed that screening would increase the diagnosis of depression by 50 % for individuals with major depression (from 45 % to 68 %). Treatment effects of diagnosed depression were then based on a meta-analysis of randomized controlled trials. Compared with no screening, periodic or annual screening of 40-years old adults resulted in rather unfavourable cost-utility ratios (US$ 68,000/€ 60,000 to US $ 260,600/€ 220,000 per QALY). The most favourable ratios (US$ 34,000/€ 30,000 to US $ 60,000/€ 44,000 per QALY) were found with one-time screening compared to no screening, yet the results were not very robust in sensitivity analysis. According to the authors, the cost-effectiveness of screening is likely to improve if more effective treatment is available.

Five studies looked at the economic case for suicide prevention: Sari et al. (2007) [49] analysed the net benefit of two programmes, general suicide awareness versus peer support programmes for the prevention of suicides in university students in Florida. They applied a simulation model using data on recorded suicides and a meta-analysis to estimate the effectiveness of the two prevention programmes. Based on the estimated effect rates for general education and peer support programmes to prevent suicide (57 % and 60 % respectively), the authors came to the conclusion that implementing both programmes in all universities in Florida would result in net benefits of US$ 21 million (€ 18 million) and US$ 32 million (€ 27 million) respectively, representing benefit-cost ratios of US$ 2.36:1 and US$ 4.30:1 (€ 2:1 and € 3.70:1).

Zaloshnja et al. (2003) [44] retrospectively examined the cost-effectiveness of a suicide prevention programme for adolescent Native Americans belonging to the Western Athabaskan tribe in rural New Mexico. A wide range of interventions were included in the programme including the use of lay people trained as 'natural helpers' to support young people in crisis and notify mental health professionals of the need for assistance. The programme was evaluated by a mirror study comparing the suicide rate of the targeted age group before and after the intervention. The suicide rate of other age groups was used as a 'quasi' control group. Economic evaluation was conducted by means of cost benefit and cost utility analysis. Results of the former indicated a cost-benefit ratio of US$ 47 (€ 40) per $ invested while the latter revealed a very favourable incremental cost effectiveness ratio of US$ 460 (€ 514) per QALY gained. The study is however limited by the lack of an appropriate control group and the fact that it was impossible to attribute any change in the suicide rate to a specific element of the programme. The very specific setting and population group for this study also implies that its results cannot be generalised easily to other contexts or settings.

Appleby et al. (2000) [42] analysed the costs and effects of an educational intervention aimed at front-line health professionals enabling them to assess and manage suicidal patients. Based on effectiveness data (which indicated high rates of programme uptake, improvement of assessment skills, as well as satisfaction with the training) they estimated cost effectiveness ratios, using different assumptions about the potential reduction in suicide rates as result of this educational intervention. They estimated that if 2.5 % of the suicides in a defined English district could be prevented by the programme, then the cost per life year gained (LYG) would be US$ 6,200 (€ 5,300) or US$ 183,000 (€ 156,200) per suicide prevented. The main limitations of the study however are that it is not based on an experimental study design and moreover that the reduction of suicide rates related to the use educational programme must be based on assumptions.

Byford et al. (1999) [41] in England analysed the cost-effectiveness of a home-based social work intervention for children and adolescents that had deliberately poisoned themselves. The programme aimed at reducing suicidal ideation and costs from further service demand. The study found no statistically significant differences between intervention and non-intervention group in terms of outcome and total costs (although the intervention group had higher intervention costs this was offset by lower costs for subsequent health and social care service utilisation). According to the authors, the intervention could be considered as cost-effective as routine care alone.

Rutz et al. (1992) [45] conducted a partial cost-benefit analysis where they compared a Swedish educational programme for general practitioners which aimed at preventing suicide via improved treatment of depression with no such intervention. As a proxy to calculating monetary benefits they used suicides prevented. The related numbers of reduced days of sick leave (10,898 days) and lives saved (19), as well as reduced drug costs, resulted in an average net benefit of US$ 37 million (€ 31.6 million). However the results were sensitive to the method of calculating monetary benefits and assumptions concerning suicides avoided. Furthermore, the analysis is limited as effectiveness data on the educational programme for suicide reduction were not derived from a rigorous experimental study design but from quasi-randomisation with retrospective comparison of two Swedish regions with and without the programme. The long term impacts of the training intervention on physician practice also remain unclear. Thus, whether any reduction in suicide can be causally related to the educational programme remains ambiguous.

### Programmes that address overall risk factors

A further group of studies evaluated interventions that were targeted at young parents or mothers having to deal with stressful socio-economic circumstances. Two programmes to support mothers in deprived London inner-city areas have been compared to standard care in terms of maternal and child health (including mental health) and costs by Wiggins et al. (2004) [40]. Both interventions were more costly than standard services and did not show any significant impact on primary outcomes. This is similar to a study by McAuley et al. (2004) [43] which evaluated the outcomes and costs of 'home-start support for young families under stress' in Northern Ireland and southern England. Again, no significant improvements in outcomes were found, but higher costs were incurred by the intervention group.

In contrast to studies identified in the UK, several US studies reported quite favourable results for ECD programmes. Most of these studies do not report outcomes for mental well-being per se, but they have been included in the analysis because they address well-accepted risk factors for mental disorders.

In the United States Schweinhart et al. (2005) [48] evaluated the monetary net benefits for pre school-support for children with low IQs from families with low socio-economic status. To do so he used effectiveness data from a specific pre-school programme ('Perry Pre School Programme'). Compared to the control-group, net benefits in a long-term evaluation arising from better school performance, higher income, reduced crime, fewer drug problems and reduced use of anti-depressants were shown. The results could, however be overestimated as the intervention was described as a pilot programme where the teachers involved were particularly committed to the programme.

Lynch (2004) [36] also used the 'Perry Pre-School study', together with three other studies that had similar aims, to calculate the long-term net benefit from the perspective of the tax payer and the public purse. According to his results, if provided to 20% of all US three and four year old children living in poverty, the programmes would result in benefit-cost ratios between US$ 4.01:1 and US$ 9.27:1 (€ 3.42:1 and € 7.91:1), depending on the different programmes' effects. Furthermore, he argued that after 17 years, the net effect on the government's budget from rolling out one of the programmes, 'Perry Preschool', on a nationwide basis would be positive. Despite this finding, generalising the results from a regionally developed and implemented programme to a broader context where educational systems may differ dramatically, raises issues of uncertainty, the more so, as this programme was developed in the 1960s and may be less appropriate to the twenty first century.

The final study identified was a simulation study by Aos et al. (2004) [37]. They quantified the effects found in scientific literature on ECD and adolescent support programmes, made some adjustments for the quality of effectiveness data and estimated the monetary benefits and costs for the selected studies. The results illustrate that from 61 selected interventions net benefits could be shown for almost two thirds. The results range from a net benefit of US$ 33,100 (€ 28,000) to net costs of US$ 52,000 (€ 44,000). The highest net benefits were demonstrated for juvenile offender programmes. Additionally, evidence for net benefit was presented for some home visiting programmes that were targeted at high-risk and/or low-income mothers. Each programme area that was examined had some interventions that were not cost effective. In addition to the limitations described for the previous examples, a further restriction of this analysis was that studies where outcomes could not be monetised were excluded.

## Discussion

With regard to quality criteria for health economic evaluations [25,34] only a few of the reviewed studies provide strong evidence that preventive interventions are cost-effective. The clearest evidence, albeit in a US context, suggests that early intervention programmes for children and adolescents are worth financing [36,37,48].

By contrast, despite the fact that the reduction of suicide is often one of the few defined targets for population mental health in many countries, there remain few studies to date that look at the cost effectiveness of suicide prevention programmes. In many respects this is not surprising, as comparatively few interventions have been the subject of effectiveness analysis [50]. This is one area where work is clearly needed.

While the number of studies identified in this area remains very limited, some analysis now suggests that the potential cost effectiveness may compare very favourably with those for other public health interventions if strategies can be demonstrated to be effective in averting suicides [51]. This may remain the case even if a conservative approach to costs is adopted, focusing solely on the immediate direct costs of suicide and future lost productivity costs averted, rather than also including the intangible costs to individuals of the immediate and unexpected loss of life that account for as much as 70% of the costs of suicide in some studies [52].

### Challenges in generating and assessing effectiveness

The variability in the quality of all studies identified is marked, although given the extra challenges, the majority of evaluators tried to develop appropriate, pragmatic and creative study designs. As with other areas of public health evaluation, the principal challenges do not relate to economic evaluation per se, but rather to the generation and attribution of effectiveness data for complex interventions with long-term outcomes.

Although the knowledge about risk factors for mental disorders has grown rapidly [5,11-15,53,54], there is no single risk factor which can explain more than 15% of the onset of a mental disorder [15]. Consequently, the detection of statistically significant risk reduction in poor mental health by an intervention may require very large sample sizes; something that is not practical or financially feasible in most studies. Effectiveness may, however be increasingly detected in interventions that are directed at several risk factors in high risk populations [55]. A similar problem arises with suicides. Completed suicides are in absolute terms relatively rare events in most countries, making it difficult to demonstrate the impact of intervention on suicides at national level, let alone at a sub national level.

Moreover, as with many other public health interventions, the long time frame between intervention and effect is a major barrier to assessing the long term effectiveness of interventions, although judicious use of modelling may help at least to look at potential cost effectiveness of interventions if different levels of effect are achieved. Nonetheless, greater investment in long-term follow up studies is required to overcome this problem.

Despite this limitation, there is an evidence base indicating that many severe mental disorders which appear in late adolescence or early adulthood may be associated with risk factors that manifest themselves in early childhood [e.g. [13,56,57]]. While long term 'Framingham studies' in mental health remain rare, there are several studies that have monitored the health and socio-economic circumstances of cohorts of children over several decades in countries including England [7], New Zealand [10] and the United States [8]. These are now being used to illustrate that the absence of intervention in childhood for mental health problems may have long standing and profound socio-economic consequences in adulthood. While these studies remain relatively rare, and moreover were not intended originally to identify mental health problems in childhood, they can be helpful in a policy context. Opportunities for more prospective evaluation may now arise [58].

### Generalisability of findings

Another challenge, given the limited number of studies available, is the extent to which the results from the evaluations may be of use in other countries or even in different regions within the same country. Due to the specific programme context of many interventions, the scope for generalisability and transferability may be restricted. The effectiveness of two identical mental health promotion or prevention programmes may vary considerably depending on the context and setting where the intervention is implemented. This is related to the cultural, social and economic context of the study population which can have a significant influence on the intervention process and its effect. As has been shown earlier, examples include those home-visiting programmes targeted at low-income mothers which have shown favourable results in the US but failed to show benefits in the UK. This also indicates the importance of gathering qualitative data on the process by which interventions are delivered which should help understand what factors lead individuals to make use of health promoting interventions. Ideally this could be undertaken concurrently with economic evaluation and may also help policy makers elsewhere both identify and estimate the costs of adaptations required to implement interventions in their own jurisdictions.

### Limitations of the review

The small number of studies identified as part of this review may, to some degree, be an artefact of the difficulty in conducting a review of economic evaluations in the areas of health promotion and public health. This has been highlighted previously [59,60]. Designing a search strategy that has a high degree of precision, that is it will find a high proportion of relevant studies, is difficult to achieve without making use of a wide range of terms related to public health and health promotion. The number of irrelevant papers in the total recall rate, that is the total number of studies identified, in our study was high, although in absolute terms the 398 studies initially identified is a modest number compared to many reviews in more medical areas. This low number of studies might also reflect the misclassification of economic terms in databases, which may mean that bone fide economic evaluations are overlooked [61].

Our exclusion criteria and review protocol also would have excluded studies which address mental health as part of general health promoting strategies, but do not distinguish between mental and physical health. Examples for this are the numerous workplace health promotion programmes that do not address mental health directly but still may have a positive impact on mental health. In addition, our reliance on the use of abstracts to determine whether or not to include or exclude studies might also mean that relevant studies might have been inadvertently excluded. Despite these limitations, our lack of economic workplace mental health promotion studies for instance, is consistent with other reviews in this area, which have reported few specific mental health orientated programmes, although stress reduction programmes are increasingly forming an element of workplace health promotion programmes [62].

## Conclusion

MHP and MDP interventions have a high potential to be of economic benefit to society. The evidence base at present is scant, making it difficult to formulate general recommendations on how to prioritise between individual interventions. Moreover, information to aid in the transferability of available results to different contexts and settings is limited. More country and population specific economic evaluations are required to strengthen this evidence base. Some of this, inevitably will mean commissioning new studies, which may take some time to deliver results; those who fund effectiveness evaluations might be encouraged to include economic analysis in their terms of reference for prospective applicants.

In the short term, another way of developing the evidence base, would be to retrospectively conduct economic evaluations making use of existing effectiveness data and attaching local estimates of resource use and costs to these interventions. This practice is common in other areas of economic evaluation, and some initiatives have been started to apply it in the area of MHP and MDP. Again, despite all their limitations, careful use of economic models might also help to provide some indication of the potential long term impact and economic consequences of interventions.

It will also be important when looking, not just at MHP or MDP, but all areas of health promotion and public health, that innovative evaluation designs are used. Conventional health economic evaluation does not typically consider issues governing the uptake and appropriateness of interventions to different populations. It is important to build into evaluation an assessment of the costs and resources required to implement interventions in different settings, cultures and contexts, and to obtain qualitative information on the success and obstacles to implementation. This type of analysis does not typically fall within the provenance of health economists and emphasises the need for a multi-disciplinary approach to evaluation. Much work remains to be done in order to gain more evidence as well as to foster methodological development.

## Competing interests

The researchers declare their independence of funding and declare that they have no competing interests.

## Authors' contributions

IZ had the main responsibility for the research design and drafting of the manuscript. All authors have made substantial contributions to conception and design as well as to study selection and study analysis. All authors read, contributed to and approved the final manuscript. The MHEEN group made substantial contributions to study identification and methodological discussions.

## Appendix: Excluded references after full-text retrieval

No full economic evaluation (n = 10):

1. Wolfersdorf M, Martinez C: **Suizid bei Depression. Verlorene Lebensjahre und Bruttosozialprodukt. Was bringt die Suizidprävention? ***Psychiatrische Praxis *1998, **25:**139–141.

2. Oberender P, Zerthh J: **Suizid und Gesundheitsökonomie: Rentiert sich eine Suizidprävention? **In *Suizidforschung und Suizidprävention am Ende des 20 Jhdts*. Edited by Wolfersdorf M, Franke C. Regensburg: Roderer; 2000

3. Smith JL, Rost K, Nutting PA, Libby AM, Elliott CE, Pyne JM: **Impact of Primary Care Depression Intervention on Employment and Workplace Conflict Outcomes: Is Value Added? ***The Journal of Mental Health Policy and Economics *2002, **5:**43–49.

4. Chatterji P, Caffray C, Crowe M, Freeman L, Jensen P: **Cost Assessment of a School-Based Mental Health Screening and Treatment Program in New York City**. *Mental Health Services Research *2004, **6:**155–166.

5. Knapp M, Barrett B, Byford S, Hallam A, Davis H, Tsiantis J, Puura K, Ispanovic-Radojkovic V, Paradisiotou A: **Primary Prevention of Child Mental Health Problems using Primary Health Care Professionals. Cost Comparisons**. *International Journal of Mental Health Promotion *2005, **7:**95–102.

6. Pelletier KR, Lutz R: **Healthy People – Healthy Business: A Critical Review of Stress Management Programs in the Workplace**. *American Journal of Health Promotion *1998, **2:**5–12.

7. Smit F, Ederveen A, Cuijpers P, Deeg D, Beekman A: **Opportunities for Cost-effective Prevention of Late-Life Depression**. *Archives of General Psychiatry *2006, **63:**290–296.

8. Price RH, Van Ryn M, Vinokour A: **Impact of a Preventive Job Search Intervention on the Likelyhood of Depression Among the Unemployed**. *Journal of Health and Social Behaviour *1992, **33:**158–167.

9. Bödeker W, Kreis J: **Der ökonomische Nutzen betrieblicher Gesundheitsförderung**. *Prävention *2002, **4:**106–109.

10. Warner KE, Wickizer TE, Wolfe RA, Schildroth JE, Samuelson MH: **Economic Implications of Workplace Health Promotion Programs: Review of the Literature**. *Journal of Occupational Medicine *1988, **30:**106–112.

Prevention of alcohol dependency (n = 4):

1. Gomel M, Wutzke SE, Hardcastle DM, Lapsley H, Reznik RB: **Cost-Effectiveness of Strategies to Market and Train Primary health Care Physicians in Brief Intervention Techniques for hazardous Alcohol Use**. *Social Science and Medicine *1998, **47:**203–211.

2. Lindholm L: **Alcohol advice in primary health care. Is it a wise use of resources? ***Health Policy *1998, **45:**47–56.

3. Chisholm D, Rehm J, Van Ommeren M, Monteiro M: **Reducing the Global Burden of Hazardous Alcohol Use: A Comparative Cost-Effectiveness Analysis**. *Journal of Studies on Alcohol *2004, **65:**782–793.

4. Deitz D, Cook R, Hersch R: **Workplace Health Promotion and Utilization of Health Services**. *The Journal of Behavioural Health Services & Research *2005, **32:**306–319.

Too general/no focus on MHP/MDP (n = 2):

1. Pelletier KR: **A Review and Analysis of the Clinical- and Cost-effectiveness and Disease Management Programs at the Worksite: 1998–2000 Update**. *American Journal of Health Promotion *2001, **16:**107–116.

2. Pelletier KR: **A Review and Analysis of the Health and Cost-Effective Outcome Studies of Comprehensive Health Promotion and Disease Prevention Programs**. *American Journal of Health Promotion *1991, **5:**311–313.

(Drug)therapy studies (n = 2):

1. Freemantle N, House A, Song F, Mason JM, Sheldon TA: **Prescribing selective serotonin reuptake inhibitors as strategy for prevention of suicide**. *British Medical Journal *1994, **309:**249–253.

2. Zhang M, Rost K, Fortney JC, Smith GR: **A Community Study of Depression Treatment and Employment Earnings**. *Psychiatric Services *1999, **50:**1209–1213.

## Pre-publication history

The pre-publication history for this paper can be accessed here:


